# Debranching abdominal aortic hybrid surgery for aortic diseases involving the visceral arteries

**DOI:** 10.3389/fcvm.2023.1219788

**Published:** 2023-07-13

**Authors:** Xiantao Ma, Yi Feng, Mbenkum Achiri Tardzenyuy, Bo Qin, Qiangzhang Zhu, Wajeehullahi Akilu, Shiliang Li, Xiang Wei, Xiang Feng, Cai Cheng

**Affiliations:** ^1^ Division of Cardiothoracic and Vascular Surgery, Tongji Hospital, Tongji Medical College, Huazhong University of Science and Technology, Wuhan, China; ^2^ Department of Cardiothoracic Surgery, Taikang Tongji (Wuhan) Hospital, Wuhan, China; ^3^ Division of Urology, Changhai Hospital, Naval Medical University, Shanghai, China

**Keywords:** aortic disease, visceral artery, thoracoabdominal aortic replacement, endovascular aortic repair, hybrid surgery

## Abstract

**Objective:**

Aortic diseases involving branches of the visceral arteries mainly include thoracoabdominal aortic aneurysm (TAAA), aortic dissection (AD) and abdominal aortic aneurysm (AAA). The focus of treatment is to reconstruct the splanchnic arteries and restore blood supply to the organs. Commonly used methods include thoracoabdominal aortic replacement, thoracic endovascular aortic repair and hybrid approaches. Hybrid surgery for aortic disease involving the visceral arteries, consisting of visceral aortic debranching with retrograde revascularization of the celiac trunk and renal arteries and using stent grafts, has been previously described and may be considered particularly appealing in high-risk patients. This study retrospectively analyzed recorded data of patients and contrasted the outcomes with those of a similar group of patients who underwent conventional open repair surgery.

**Methods:**

Between 2019 and 2022, 72 patients (52 men) with an average age of 61.57 ± 8.66 years (range, 36–79 years) underwent one-stage debranching abdominal aortic hybrid surgery. These patients, the hybrid group, underwent preoperative Computed Tomographic Angiography (CTA) and had been diagnosed with aortic disease (aneurysm or dissection) involving the visceral arteries and were at high risk for open repair. The criteria used to define these patients as high-risk group who are in the need of hybrid treatment were American Society of Anesthesiologists (ASA) class 3 or 4. In all cases, we accomplished total visceral aortic debranching through a previous visceral artery retrograde revascularization with synthetic grafts (customized Y or four-bifurcated grafts), and aortic endovascular repair with one of two different commercially produced stent grafts (Medtronic® and Lifetech®). In some cases, we chose to connect the renal artery to the artificial vessel with a stent graft (Viabahn) and partly or totally anastomosed. We analyzed the results and compared the outcomes of the hybrid group with those of a similar group of 46 patients (36 men) with an average age 54.15 ± 12.12 years (range, 32–76). These 46 patients, the conventional open group, were selected for having had thoracoabdominal aortic replacement between 2019 and 2022.

**Results:**

In the hybrid group, 72 visceral bypasses were completed, and endovascular repair was successful in all cases. No intraoperative deaths occurred. Perioperative mortality was 2.78%, and perioperative morbidity was 9.72% (renal insufficiency in 1, unilateral renal infarction in 5, Intestinal ischemia in 1). At 1-month postoperative CTA showed 2 endoleaks, one of which was intervened. At follow-up, there were unplanned reoperation rate of 4.29% and 5 (7.14%) deaths. The remaining patients’ grafts were patent at postoperative CTA and no endoleak or stent graft migration had occurred. In the conventional open group, 1 died intraoperatively, 4 died perioperatively, perioperative mortality was 10.87% and complications were respiratory failure in 5, intestinal paralysis/necrosis in 4, renal insufficiency in 17, and paraplegia in 2. At follow-up, 5 (12.20%) patients presented with synthetic grafts hematoma 4 (9.76%) patient died, and 6 (14.63%) patients required unplanned reoperation intervention.

**Conclusion:**

Hybrid surgery is technically feasible in selected cases. For aortic diseases involving the visceral arteries, the application of hybrid abdominal aorta debranching can simplify the operation process, decrease the risks of mortality and morbidity in high-risk and high-age populations and decrease the incidence of various complications while achieving ideal early clinical efficacy. However, a larger series is required for valid statistical comparisons, and longer follow-ups are necessary to evaluate the long-term efficacy of hybrid surgery.

## Introduction

The visceral arterial branches mainly involve the renal arteries, the celiac trunk artery and the superior mesenteric artery (1). Aortic diseases involving branches of the visceral arteries mainly include thoracoabdominal aortic aneurysm (TAAA), aortic dissection (AD) and abdominal aortic aneurysm (AAA) (2–4). A common feature of a classic TAAA, as well as some diseases such as AD and AAA, is that the diseases may involve the visceral arteries and, in the surgical procedure, reconstruction of these visceral arterial branches is possible. Currently, the main modalities for the treatment of abdominal aortic disease are thoracoabdominal aortic replacement, endovascular aortic repair (EVAR) and hybrid approaches (5).

Since its first description in 1955, open repair has been considered the gold standard of treatment for aortic diseases and remains among the most difficult surgical approach due to the associated vital structures, such as the mesenteric/renal branches and the segmental arteries involved in the lesion (6). For aortic diseases involving the visceral artery branches, it is necessary to reconstruct the visceral artery branches while treating the aortic disease (7). Due to the long operation time and extensive trauma, various serious perioperative complications, such as internal organ ischemia, massive intraoperative hemorrhage, paraplegia, renal failure, and infection, are possible and the risk of mortality is high (8, 9).

Another alternative approach to traditional open repair is EVAR with endograft techniques (10). There is no doubt that a total endovascular approach would benefit patients with aortic diseases, but individualized stents may take years to become widely available (11). Treatment modalities consisting of visceral parallel graft (chimney/snorkel) techniques with off-the-shelf devices have a greater probability of causing complications such as endoleaks and displacements, so EVAR is now more beneficial for treating subrenal aortic diseases (12, 13). Three-dimensional, laser and customized branch/bifurcation stent grafts are now available for the treatment of TAA (14–17).

Hybrid approaches consisting of visceral aortic debranching with retrograde revascularization of the mesenteric, coeliac trunk and renal arteries and aortic lesion exclusion using commercially available stent grafts have been described in several reports (18, 19). This study analyzed recorded data of a series of high-risk patients with a history of aortic surgery who underwent hybrid repair for aortic diseases at some institutes in the last 4 years and compared the outcomes in this group with those in a similar group of patients who underwent conventional open repair for aortic diseases. The aim of this paper is to introduce a relatively less invasive surgical approach for patients who may have had total thoracoabdominal aortic replacement in the past.

## Patients and methods

**Figure 1:**
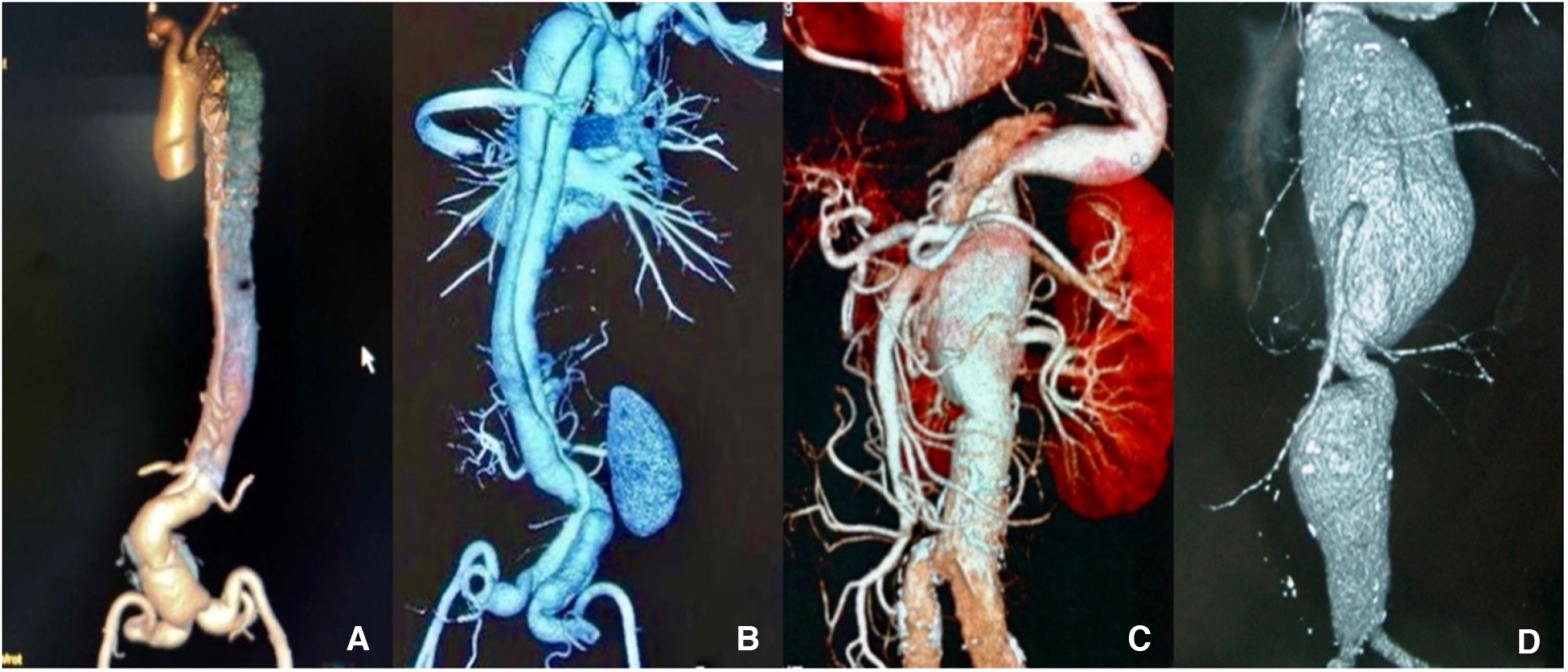
(**A,B**) Preoperative computed tomography angiography (CTA) scan shows thoracoabdominal aortic dissection (TAAD). (**C,D**) CTA shows thoracoabdominal aortic aneurysm (TAAA). The images show that all lesions of the aorta involve the visceral arteries.

Between 2019 and 2022, 72 patients (52 men) with a mean age of (61.57 ± 8.66) years (range, 36–79 years) underwent a one-stage debranching abdominal aortic hybrid procedure at Tongji Hospital, Tongji Medical College, Huazhong University of Science and Technology, Changhai Hospital, Naval Medical University, and other centres. These patients, the hybrid group, underwent preoperative Computed Tomographic Angiography (CTA) (Figure 1) and had been diagnosed with aortic disease (aneurysm or dissection) involving the visceral arteries. The group was assessed preoperatively as being at high risk and the criteria used to define these at-risk groups in need of hybrid surgery were American Society of Anesthesiologists (ASA) Level 3 or 4. At the same time, we selected a similar group of 46 patients (36 men) who had undergone thoracoabdominal aortic replacement between 2019 and 2022 as the conventional open group, whose mean age was (54.15 ± 12.12) years (range, 32–76 years). We analyzed the results and compared these results with the hybrid group. The aim of this study was to describe how to use the hybrid procedure in high-risk and high-age populations.

### Hybrid group

Beginning in 2019, 72 high-risk patients underwent one-stage stent graft repair with visceral aortic debranching and prior retrograde revascularization of visceral arteries. The criteria we used to define these patients as being at high risk for conventional repair were American Society of Anesthesiologists (ASA) class 3 or 4.

All patients underwent a one-stage procedure performed in the operating room. All patients were operated on under general anesthesia with tracheal intubation. The abdominal aorta, common iliac arteries, and the first 2 cm from the origin of the common hepatic artery, superior mesenteric artery, and renal arteries were exposed, when needed, through a transperitoneal midline approach with the patient in a supine position. Synthetic grafts with diameters of 6 or 8 mm and 10 were used in all patients. We preferably used customized Y grafts or separated bypass grafts, or both, and four-bifurcated grafts for each recipient vessel. In some cases, we accomplished total visceral aortic debranching through a previous visceral artery retrograde revascularization with four-bifurcated grafts.

There are two inflow site options: the prograde route (diversion from the ascending aorta) and the retrograde route (diversion from the iliac artery and distal abdominal aorta). We generally choose the retrograde route. The choice of inflow site for retrograde visceral artery bypass grafting was based on the extent of the lesion, the presence of prior abdominal aortic repair, and the quality of the walls of the native aorta and iliac arteries. The proximal segment of this iliac artery was blocked, an incision of approximately 10 mm was made in the wall of the iliac artery, and this incision was continuously anastomosed end-to-side with the opening of the two-branch artificial vessel. Each stitch needs to be tightened to avoid twisting, stenosis and anastomotic leakage due to improper placement of the artificial vessel.

**Figure 2:**
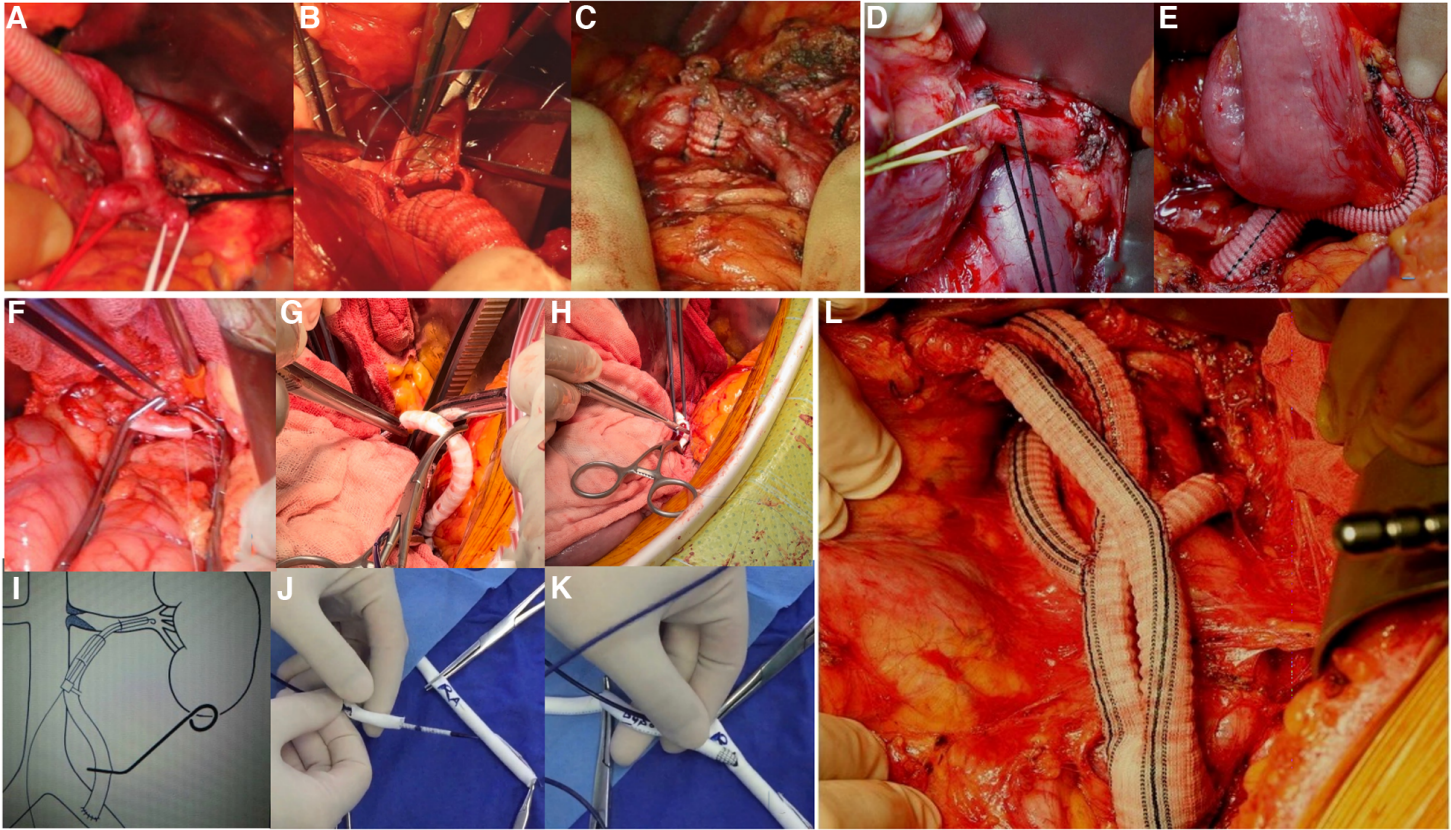
(**A–C**) Intraoperative photographs show details of the reconstructed celiac trunk. (**D,E**) Intraoperative photographs show the reconstruction of the superior mesenteric artery. (**F**) The photograph shows an end-to-side anastomosis of the renal artery. (**G,H**) Intraoperative photographs show a Viabahn was used to connect the graft to the renal artery, secured with sutures on the outside of the graft. (**I**) Diagram of the reconstructed renal artery with Viabahn. (**J,K**) Demonstration of the reconstructed renal artery with Viabahn. A guidewire is introduced into the pre-sutured graft and the Viabahn is advanced. An opening is made in the anterior wall of the renal artery and the guidewire is introduced into the distal end of the renal artery. Viabahn is then advanced through the guidewire into the renal artery for release. (**L**) Intraoperative photograph shows the reconstruction of the visceral arterial branches is finished.

Reconstruction of the outflow pathways allowed reconstruction of the visceral arterial branches. All the donor vessels were anastomosed end-to-side (Figure 2).

For celiac trunk revascularization, the graft was routed through the middle of the omental sac, either behind or in front of the pancreas, an arteriotomy was made in the common hepatic artery or the splenic artery, and an end-to-side anastomosis was made. If the proximal abdominal trunk is not tied, a type 2 endoleak can develop, so it is essential to prevent spinal cord ischemia and eliminate the need for cerebrospinal drainage to the same extent as in the open branch technique.

An end-to-side anastomosis of the superior mesenteric artery was made next to the flexor ligament. During the operation, part of the flexor ligament should be preserved, the duodenum should not be excessively dissected, and the formation of an acute angle between the bridge vessels and the superior mesenteric artery should be avoided after mesenteric repositioning.

When anastomosis of the renal artery was required for reconstruction, the kidney was wrapped in ice slush before and during renal artery cross-clamping and the temperature was lowered from 15°C to 18°C. An end-to-end anastomosis was performed when the renal artery was deep, and an end-to-side anastomosis was performed when the renal artery was shallow. To save time in reconstructing the renal artery, we usually used a Viabahn (GORE®) with a diameter of 6 or 7 mm and a length of 10 cm (depending on the diameter of the involved renal artery as measured by the preoperative CTA) that connected the artificial vessel to the renal artery. Renal warm ischemia time was limited to 2 min with Viabahn, so ice slush was not used for renal hypothermia (20). A bypass graft (6 mm in diameter) was sutured to the Y graft, which underwent end-to-side anastomosis with the iliac artery. A guidewire was introduced into the presutured graft over which the Viabahn was advanced. An opening was made in the anterior wall of the renal artery, and the guidewire was introduced distal to the renal artery. The Viabahn was then advanced through the guidewire into the renal artery to a certain distance as measured by preoperative CTA. A silk knot or suture was used to secure the stent to the outside of the artificial vessel.

In all the reconstructions, the grafted vessels were ligated proximally to prevent retrograde perfusion of the sac after endovascular exclusion of the aneurysm. The grafts were then covered with retroperitoneum or an omental flap whenever possible.

**Figure 3:**
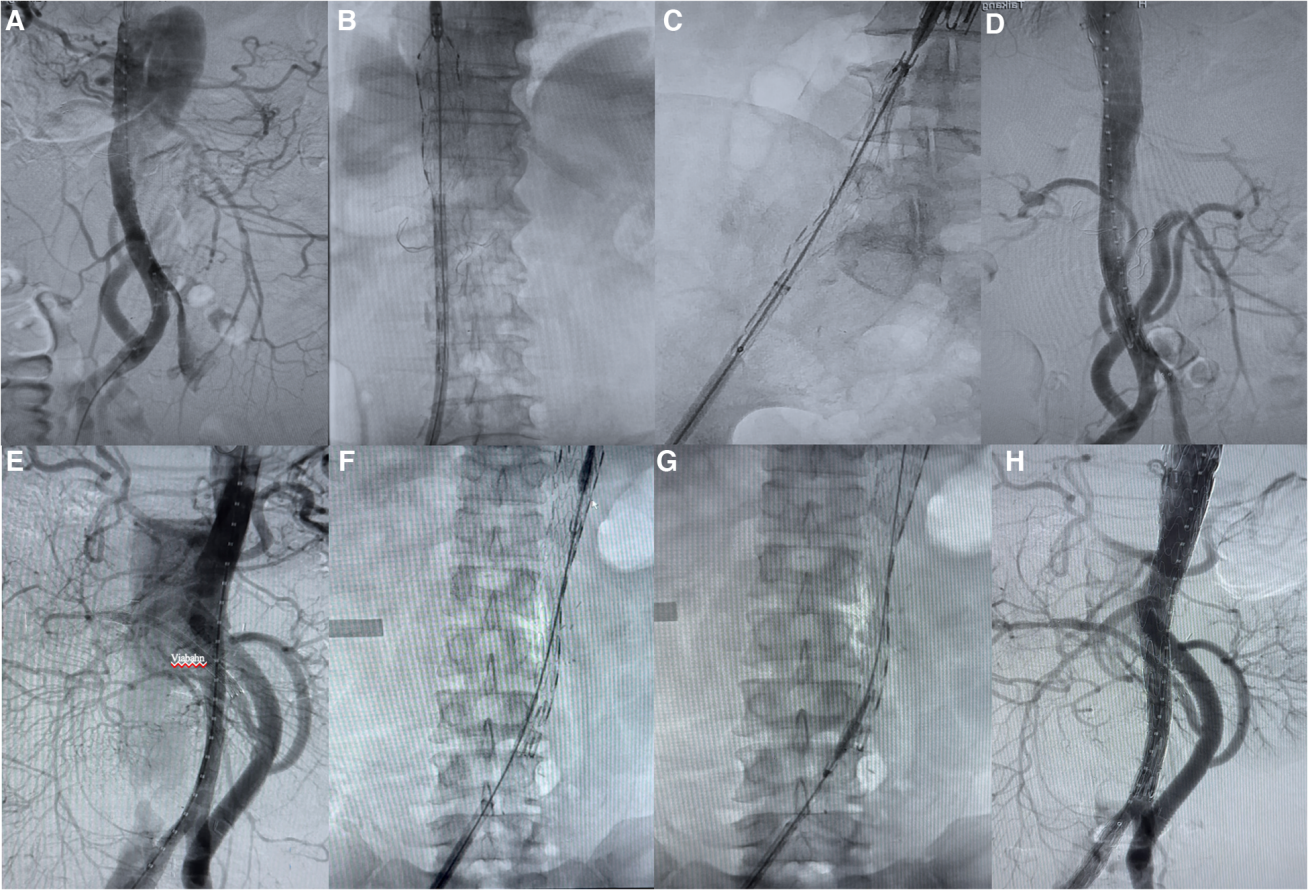
Complete endovascular repair of aortic disease. (**A–D**) Renal arterial reconstruction without Viabahn. (**E–H**) Renal arterial reconstruction with Viabahn.

The access vessel for endograft insertion was the common femoral artery (exposed through an inguinal incision). Two stent grafts (Medtronic® and Lifetech®) were used, and 1–3 stent grafts were deployed in each patient. Complete aortography was performed after deployment of the endografts (Figure 3).

All patients were transferred to the intensive care unit (ICU) after the operation. Antiplatelet therapy (aspirin) was begun on the first postoperative day and was maintained for at least 3 months. Sensation and movement of the lower extremities, postoperative morbidity and short-term mortality were recorded. CTAs were performed at 3, 6, or 12 months after the operation in all the survivors and were carefully compared with the preoperative total CTAs to evaluate renal artery patency, to assess the stent and bypass grafts, and to identify renal infarction, bleeding at the anastomosis, and stent-grafter elated complications, such as migration, kinking, or fracture.

### Conventionally treated group

From the entire series of patients who had undergone thoracoabdominal aortic replacement after 2019, we selected 46 patients (36 men) who had undergone prior aortic surgery (19.57% ascending, 15.22% descending, and 2.17% abdominal aortic repair) and had an ASA classification of 3 or 4.

All procedures were performed electively. Cell salvage, rapid infusers, and transfusion were used. All patients were operated on under general anesthesia, tracheal intubation and respiratory ventilation.

The surgery was performed from the left posterolateral 6th or 7th intercostal space to the inferior border of the costal arch and continued to the left rectus abdominis muscle. Depending on the extent of the lesion, the surgery can extend to the iliac fossa, allowing the opening of the diaphragm to expose and explore the thoracoabdominal aorta. Distal aortic perfusion with left atrial femoral artery bypass or hypothermic visceral perfusion with or without cerebrospinal fluid drainage was routinely used (21). Four branches of aortic grafts with diameters of 26 and 28 mm (branch diameter of 8 or 10 mm) were used in all patients. Reconstruction of the proximal aorta, visceral artery branches, and distal aorta was accomplished after sequential segmental block. End-to-end anastomosis was performed between these arteries and the graft. In some patients, the arterial wall containing the opening of the celiac trunk, superior mesenteric artery, and right renal artery could be directly anastomosed to the sidewall of the graft, and an end-to-side anastomosis of the left renal artery was performed separately. The patent intercostal arteries below the 8th thoracic vertebra were reconstructed, and the remaining occluded intercostal arteries were sutured. In cases of dissection/aneurysm involving the abdominal aortic bifurcation and below, the graft branches were anastomosed downward to the iliac artery.

Patients were evaluated with postprocedural CTA at 3, 6 and 12 months and yearly thereafter. Clinical follow-up was also conducted every 6 months. We analyzed the outcomes in our patients, reporting the results and methods.

## Results

### Hybrid group

Debranching abdominal aortic hybrid surgery of aortic diseases involving visceral arteries in all 72 patients. No intraoperative deaths occurred. Because we performed this procedure on some of our patients at other heart centers, we lost some of the patient data from the procedure. By analyzing the perioperative data of 72 patients, we mainly analyzed the incidence of postoperative complications and mortality.

**Figure 4:**
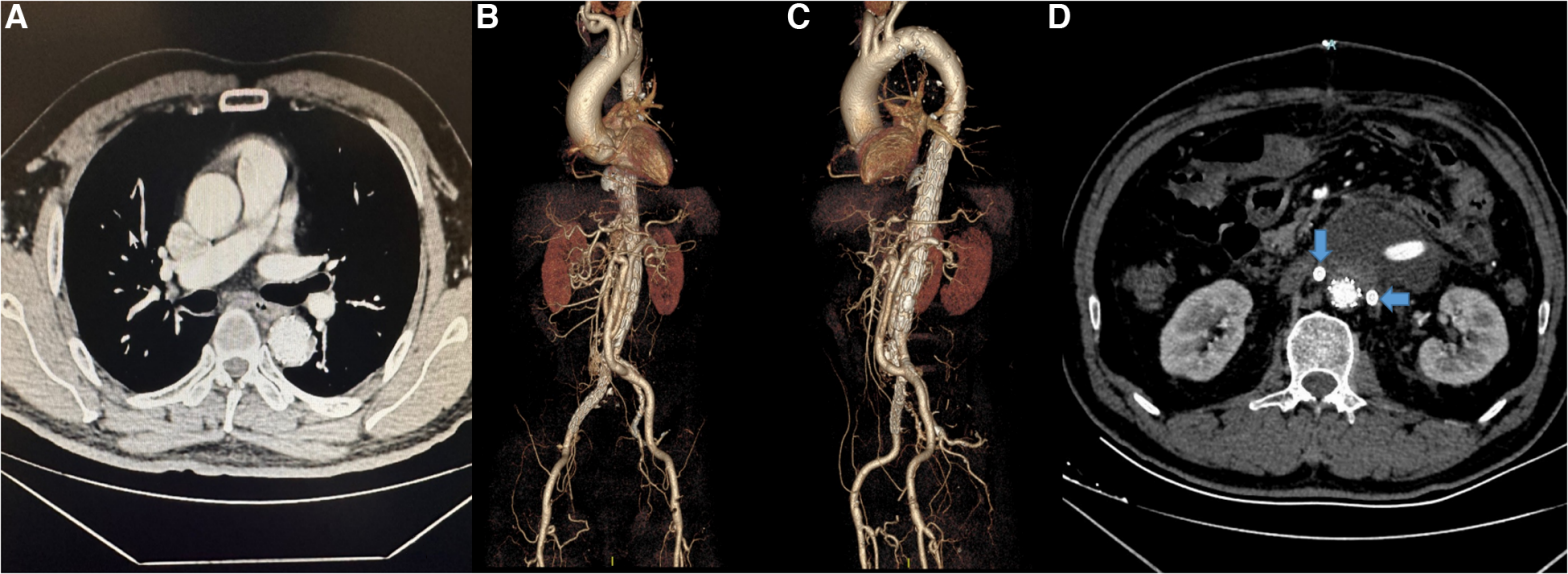
(**A–C**) The CTA and 3-dimensional reconstruction of patient after surgery. (**D**) The bilateral renal arteries (arrows) were patent.

The hybrid repair was clinically successful in 70 (97.22%) of 72 patients (Figure 4). Two patients died postoperatively for a mortality rate of 2.78%: one related to disseminated or diffuse intravascular coagulation (DIC) and one from ruptured aneurysm. Other postoperative complications included acute renal failure in one patient (1.39%) that resolved without dialysis that was considered as occlusion of the bridge vessel on one side and failure of revascularization on the other side, 5 patients (6.94%) presented with unilateral renal infarction considered to be bridge vessel occlusion, and one (1.39%) presented with left hemicolectomy necrosis considered the inferior mesenteric artery was not revascularized because of high regurgitant pressure.

During the follow-up period patients had one bridge vessel occlusion requiring unplanned reoperation (A patient was found to have renal artery occlusion due to VB displacement during the 5-years post-operative review. After dilatation with a balloon, the renal artery was recanalized.), two endoleaks requiring unplanned reoperation management (unplanned reoperation rate of 4.29%), one pulmonary infection due to aneurysm compression, and 5 (7.14%) deaths. In total, there were even 7 (9.72%) surgical and aneurysm-related deaths.

### Conventionally treated group

One intraoperative death occurred. The mean procedure time was 398 ± 143 min. A mean of 5.4 ± 6.4 U of packed red blood cells was transfused for a median blood loss of 1852 ± 2124 ml. There were 4 postoperative deaths. Perioperative mortality was 10.87%. Perioperative complications included 2 patients with permanent paraplegia, 4 with intestinal paralysis/necrosis, 4 patients with unilateral renal infarction, 4 with venous thrombosis (one of which was a pulmonary embolism), 17 with renal failure, 7 with surgical site infection, 2 with graft infection, and 19 with pulmonary infection and 5 with respiratory failure. All patients were admitted to the intensive care unit, where the stayed for a mean of 4.30 ± 3.35 days.

At post-discharge follow-up, there were 4 (9.76%) deaths here, 6 (14.63%) patients required unplanned reoperation intervention (2 of EVAR, 2 of chest wall debridement and suturing, 1 of thoracotomy hemostasis and 1 of abdominal hemostasis), and 5 (12.20%) patients presented with synthetic grafts hematoma. The overall surgical and aneurysm-related death rate was 19.57%.

The age of the hybrid group was significantly higher than that of the conventional group, as the hybrid surgery was used preferentially on people who were at high-risk or of high-age (61.57 ± 8.66, 54.15 ± 12.12; respectively; *p *< 0.05). The rates of complication such as renal failure, respiratory failure, and lower extremity thrombosis within 30 days of hybrid surgery were also significantly lower in the hybrid surgery group than in the conventional surgery group (*p *< 0.05) (Table 1). Although there was no significant difference in mortality and unplanned reoperation rates between the hybrid and conventional treatment groups, the desired results were achieved in an older age group.

**Table 1:** Outcomes.

** **	Hybrid group	Conventionally treated group	*p*
Perioperative period			
Death	2/72 (2.78%)	5/46 (10.87%)	0.0696
Paraplegia	0/72 (0.00%)	2/46 (4.35%)	0.0743
Renal failure	1/72 (1.39%)	17/46 (36.96%)	<0.0001
Unilateral renal infarction	5/72 (6.94%)	4/46 (8.70%)	0.7267
Intestinal paralysis/necrosis	1/72 (1.39%)	4/46 (8.70%)	0.0546
Respiratory failure	0/72 (0.00%)	5/46 (10.87%)	0.0043
Deep venous thrombosis	0/72 (0.00%)	4/46 (8.70%)	0.0109
Postoperative follow-up			
Death	5/70 (7.14%)	4/41 (9.76%)	0.6264
Unplanned reoperation	3/70 (4.29%)	6/41 (14.63%)	0.0539
Surgical/aneurysm-related deaths	7/72 (9.72%)	9/46 (19.57%)	0.1277

## Discussion

The main aortic diseases involving the visceral arterial branches, including the renal arteries, celiac trunk and superior mesenteric artery, are TAAA, AD and AAA. TAAAs remains a challenging clinicopathology involving the visceral arteries, although it accounts for only 7%–15% of all aortic and peripheral aneurysms (22). TAAAs are mostly caused by aortic degeneration and have poor prognoses, with a 42%–70% probability of untreated rupture after diagnosis and an average survival time of 3 years (23). AAD refers to an aortic dissection involving the aorta below the diaphragm and is divided into two main types (24–26). The first type is an isolated abdominal aortic dissection (IAAD), which involves rupture of the abdominal aorta only and accounts for approximately 1%–4% of all aortic dissection cases. The other type is secondary thoracic aortic coarctation, which results from the continuation of the lesion into the abdominal aorta following an aortic tear, which is more common. A common characteristic of these lesions is their involvement with the visceral branch arteries, so it may be necessary to reconstruct these visceral artery branches during surgery. The choice of treatment depends primarily on the pathology of the aneurysm, the extent of the lesion and the patient's condition.

Thoracoabdominal aortic replacement is still one of the most difficult surgical approaches due to the associated vital structures, such as the visceral/renal branches and the segmental arteries involved in the lesion. First reported by Etheredge in 1955, open surgery has been the preferred treatment for this type of aortic disease, especially TAAA (6). Open surgery is not only traumatic, as it requires an incision of the chest, abdomen, and diaphragm, it also requires cardiopulmonary bypass, thereby leading to coagulation disorders (27, 28). Recent reports from high-volume aortic centers have shown that the mortality rate after open repair in young patients (younger than age 50 years) ranges from 3% to 6%, and the mortality rate in older patients remains 8%–17% (29, 30). Since Crawford proposed the inclusion technique for the treatment of TAAA in 1978, the outcome of the surgery has improved significantly due to the use of adjunctive procedures, including selective visceral perfusion, distal aortic perfusion and cerebrospinal fluid drainage (31–34). Despite advances in surgical technique, spinal cord protection and postoperative intensive care support, patients undergoing open surgery still have a 30-day mortality rate of 7%–17% (35). Fifty percent of patients have a significant risk of complications, including cardiac ischemia, pulmonary events, hemorrhage, spinal cord ischemia and acute renal failure (8, 36). Even though these outcomes are poor, selective repair remains necessary, since the 2- and 5-year mortality rates of patients who do not undergo an operation are 76% and more than 95%, respectively (37).

Another alternative approach to traditional thoracoabdominal aortic replacement is endovascular aortic repair (EVAR) with stent grafts. Using a stent graft, Parodi pioneered the minimally invasive treatment of abdominal aortic aneurysms in 1991 (38). In 1994, Dake performed a thoracic endovascular aneurysm repair to successfully treat a descending aortic aneurysm (39). EVAR can be performed percutaneously using a transfemoral method, avoiding thoracoabdominal incisions, diaphragm division, left heart or cardiopulmonary bypass and cross-clamping of the aorta. There is uninterrupted aortic flow and perfusion of the internal organs and lower limb vessels during the procedure. EVAR therefore radically reduces the physiological demands, the risk of end organ ischemia, blood loss, fluid requirements and the need for transfusion products. EVAR is a suitable treatment for older, weaker and higher risk patients who have been previously regarded as unsuitable candidates for open surgery. In the last decade, the number of EVARs has doubled, and its feasibility has already been demonstrated (40, 41). With the progress of EVAR, more abdominal aortic diseases involving the visceral arteries can be treated with these methods, including visceral parallel graft (chimney/snorkel) techniques, multibranched stent graft and fenestrated techniques (42). When the coeliac trunk, superior mesenteric and renal arteries are from a nonaneursymal implantation site, a simple hole (fenestration) in the stent graft is sufficient. When these arteries arise from the aneurysm, flow must be carried across the aneurysm through branches of the stent graft. Despite these advances, there are shortcomings and other anatomical limitations. Small vessels, such as the intercostal arteries, cannot be reconstructed by endovascular methods in most cases, raising concerns that extensive aortic coverage may increase the risk of spinal cord injury (43). Some papers have shown that compared to open surgical repair, endovascular aortic repair and subrenal endovascular repair is associated with better quality of life indicators at 1 year postoperatively (44). Three-dimensional, laser and customized branch/bifurcation stent grafts have been introduced for the treatment of TAA. These new technologies are constantly advancing to facilitate better individualized treatment (14–17). There is no doubt that a total endovascular approach would benefit patients in poor conditions (45, 46).

Hybrid surgery, a combination of open and endovascular techniques, for the treatment of aortic disease has been suggested to reduce surgical injury and improve outcomes, especially in high-risk patients, and was first reported in 1999 (47). The hybrid surgery provides sufficient anchorage for EVAR through open visceral artery reconstruction and “transfer” from the diseased aorta to the healthier aorta. Thus, hybrid surgery can be widely used in patients with paravisceral aneurysms (48). Compared to traditional thoracoabdominal aortic replacement, hybrid repair avoids the need for thoracotomy, extracorporeal perfusion, and supraceliac aortic clamping, and therefore, hemodynamics is more stable intraoperatively and there is less risk of causing an ischemia‒reperfusion injury to the visceral organs (49). It significantly decreases postoperative pain and the incidence of pulmonary complications in patients with underlying pulmonary diseases (50). The disadvantage is that it still requires laparotomy. For abdominal obesity, exposure of the superior mesenteric artery and coeliac trunk is difficult, intraoperative damage to the pancreatic duct may lead to the formation of postoperative pancreatic cysts, and damage to the portal vein may lead to postoperative bleeding. In addition, postoperative intestinal obstruction or swelling may affect the patient's respiration (51). Although hybrid repair was developed as a less invasive approach than traditional open surgery, the 30-day mortality rate after hybrid techniques in many reports is disappointingly high (e.g., operative mortality, 3.6%–12.5% and renal failure, 20%), which has been attributed to the use of the technique in patients who are unsuitable or considered to be high-risk open surgery patients (50, 52). Although hybrid surgery has been debated, its efficacy and safety in treating aortic disease has been recognized in the 2017 Clinical Practice Guidelines of the European Society for Vascular Surgery (Class IIa/C evidence) (53).

In fact, several patients selected for hybrid surgery are not suitable for open repair because they have severe medical comorbidities. Another benefit of hybrid surgery is that it can be used in emergencies or for patients with acute cases of ruptured TAAAs or TAADs with poor perfusion who have severe respiratory or cardiac disease and are unlikely to survive open repair. There are no other repair options, with current endovascular techniques, for these patients because there are no available fenestrated stent grafts for narrow true lumens, branch stents that can be easily introduced into small spaces that limit maneuverability, branch arteries fed by the false lumen, and no distal healthy anchor zone. In our surgical practice, we consider elective hybrid surgery for patients who are unable to undergo major thoracoabdominal procedures, who have connective tissue disorders and whose anatomy does not allow EVAR.

Since 2019, we have performed debranching abdominal aortic hybrid surgery on 72 patients with aortic disease involving the visceral arteries, using the Viabahn for some of them. Preoperative CTA showed that all patients had lesions involving the abdominal aorta and visceral arteries. A transabdominal abdominal approach is usually performed to expose the entire abdominal aorta and target vessels. Retrograde extra-anatomic bypass of the abdominal axis, superior mesenteric artery, and bilateral renal arteries is performed by using the iliac artery as the inflow port. In patients with aortic coarctation resulting in iliac artery disease, subrenal aortic replacement is considered. However, one clear disadvantage of anastomosis of the branches is the renal ischemia time. Standard suture anastomosis in renal arteries inevitably requires a long renal ischemic time of 25 min (54). Although hybrid surgery reduced the renal ischemic time to approximately 10 min, we found that the Viabahn reduced the renal ischemic time further by avoiding the need for a suture anastomosis and extensively exposing the renal artery (55). Our limited experience has shown that the Viabahn technique is workable and effective in all planned procedures, regardless of the anatomical location or quality of the visceral vessels.

The aims of our study were to to introduce a relatively less invasive surgical approach for patients in high-risk and high-age populations and to compare the differences between thoracoabdominal aortic replacement and hybrid surgery for aortic disease involving the visceral arteries in terms of mortality and complication rates (e.g., renal failure, unilateral renal infarction, intestinal paralysis/necrosis, respiratory failure, and deep venous thrombosis). There are very few studies that have compared these two methods. We present a hybrid surgery using the Viabahn for the treatment of the renal artery, which prevents stenosis of the renal artery anastomosis, avoids the need for ice chips, and shortens the length of renal artery decortication and the time of renal ischemia. As seen in our statistics, the rate of transient renal failure after hybridization was significantly lower. However, the rates of unilateral renal infarction and mortality at the follow-up visit following hybrid surgery were not significantly different from those following conventionally open surgery. During the follow-up period, three patients who underwent hybrid surgery underwent unplanned reoperation for reasons that included bridge vessel occlusion and endoleak.

It is evident that debranching abdominal aortic hybrid surgery also has limitations. First, endoleaks are an important cause of failure, and lesion development can lead to retrograde tearing of the dissection or enlargement and rupture of the aneurysm. Second, the current option for the inflow site is mainly a retrograde path, which has the risk of distant anastomotic stenosis and even branch occlusion. Viabahn's dislodgement or movement is one of the few undesirable occurrences. This can lead to an associated renal infarction with renal artery occlusion. Finally, the most important concern is that open repair is a major, technically challenging approach, particularly when complete visceral and/or renal revascularization is needed. The abdominal complications associated with an incision of the abdomen is a concern for doctors.

We also consider how to avoid these problems in our management. First, we ligate or suture the proximal end of the visceral artery during the debranching operations and then select an adequate anchorage as well as a stent graft of an appropriate size. Next, we performed a separation anterior to the left renal fascia and anastomosis of the synthetic grafts in the left iliac artery. The advantage of this is that the exposure is adequate, the anastomosis is more favorable, and the synthetic grafts are less prone to distortion. Finally, and most importantly, the Viabahn is used to perform a “no stitching anastomosis” of the aortic branches of the renal viscera in the debranching procedure. This tool avoids exposure and the need for anastomosis, which are technically demanding, reduces the interruption of blood and simplifies the procedure. However, we securely fix the Viabahn by suturing or tying knots from the outside where the Viabahn and the graft are in contact.

Hybrid surgery has the advantages of both EVAR and thoracoabdominal aortic replacement. The role of endovascular techniques in the treatment of aortic disease continues to develop. Hybrid surgery does not replace conventional open surgery but offers an option for high-risk patients who may be denied treatment. The long-term efficacy of hybrid surgery has yet to be elucidated, and patients still require routine surveillance to monitor for endoleak development. However, limited studies have shown results and outcomes that are favorable to hybrid surgery.

The biggest limitation of our study is the sample size of the hybrid group, as patients underwent surgery in different cities, leading to missing data and preventing a complete comparison of the metrics during surgery. The quality of the study was also limited by the asynchronous nature of the two groups, the economic status of the patients and the retrospective nature. Studies with a large series of patients with detailed medical histories and a longer follow-up are necessary to provide meaningful statistics for debranching abdominal aortic hybrid surgery for aortic diseases involving the visceral arteries and visceral artery reconstructions with the Viabahn.

## Data Availability

The raw data supporting the conclusions of this article will be made available by the authors, without undue reservation.

## References

[1] Kabbani LS Criado E Jr UG Patel HJ Eliason JL Rectenwald J Hybrid repair of aortic aneurysms involving the visceral and renal vessels. Ann Vasc Surg. (2010) 24(2):219–24. 10.1016/j.avsg.2009.08.007 19932951

[2] Cochennec F Marzelle J. Les syndromes aortiques aigus [acute aortic syndromes]. Presse Med. (2018) 47(2):140–52. 10.1016/j.lpm.2018.02.001 29526427

[3] Smith TA Gatens S Andres M Modrall JG Clagett GP Arko FR. Hybrid repair of thoracoabdominal aortic aneurysms involving the visceral vessels: comparative analysis between number of vessels reconstructed, conduit, and gender. Ann Vasc Surg. (2011) 25(1):64–70. 10.1016/j.avsg.2010.06.004 20889300

[4] Yamaguchi H Murata S Onozawa S Sugihara F Hayashi H Kumita SI. Strategy for the treatment of spontaneous isolated visceral artery dissection. Eur J Radiol Open. (2018) 6:9–15. 10.1016/j.ejro.2018.11.003 30560151 PMC6289943

[5] Rosenblum JM Chen EP. Thoracoabdominal aortic aneurysm repair: open, endovascular, or hybrid? Gen Thorac Cardiovasc Surg. (2019) 67(1):175–9. 10.1007/s11748-017-0820-y 28856583

[6] Etheredge SN Yee J Smith JV Schonberger S Goldman MJ. Successful resection of a large aneurysm of the upper abdominal aorta and replacement with homograft. Surgery. (1955) 38(6):1071–81. 10.1016/0371-1951(55)80039-2 13274266

[7] Li Y Li Z Feng J Feng R Zhou J Jing Z. A novel solution for distal dilation of chronic dissection after repair involving visceral branches: the roadblock strategy. Front Cardiovasc Med. (2022) 9:821260. 10.3389/fcvm.2022.821260 35355962 PMC8959700

[8] Khoury MK Acher C Wynn MM Acher CW. Long-term survival after descending thoracic and thoracoabdominal aortic aneurysm repair. J Vasc Surg. (2021) 74(3):843–50. 10.1016/j.jvs.2021.02.048 33775746

[9] Polanco AR D'Angelo AM Shea NJ Allen P Takayama H Patel VI. Increased hospital volume is associated with reduced mortality after thoracoabdominal aortic aneurysm repair. J Vasc Surg. (2021) 73(2):451–8. 10.1016/j.jvs.2020.05.027 32473340

[10] Patel R Sweeting MJ Powell JT Greenhalgh RM, EVAR trial investigators. Endovascular versus open repair of abdominal aortic aneurysm in 15-years’ follow-up of the UK endovascular aneurysm repair trial 1 (EVAR trial 1): a randomised controlled trial. Lancet. (2016) 388(10058):2366–74. 10.1016/S0140-6736(16)31135-7 27743617

[11] Kheirelseid EA Gardiner R Haider SN Martin Z Colgan MP O'Neill SM Endovascular repair of thoracoabdominal aortic aneurysm (TAAA): early experience. Ir J Med Sci. (2014) 183(2):153–60. 10.1007/s11845-013-0974-2 23757213

[12] Eagleton MJ Follansbee M Wolski K Mastracci T Kuramochi Y. Fenestrated and branched endovascular aneurysm repair outcomes for type II and III thoracoabdominal aortic aneurysms. J Vasc Surg. (2016) 63(4):930–42. 10.1016/j.jvs.2015.10.095 26792544

[13] Pecoraro F Pfammatter T Mayer D Frauenfelder T Papadimitriou D Hechelhammer L Multiple periscope and chimney grafts to treat ruptured thoracoabdominal and pararenal aortic aneurysms. J Endovasc Ther. (2011) 18(5):642–9. 10.1583/11-3556.1 21992633

[14] Chuter TA Gordon RL Reilly LM Goodman JD Messina LM. An endovascular system for thoracoabdominal aortic aneurysm repair. J Endovasc Ther. (2001) 8(1):25–33. 10.1177/152660280100800104 11220464

[15] Zymvragoudakis V Saha P Gkoutzios P Zayed H Abisi S. Use of the off-the-shelf pre-cannulated E-nside endograft to overcome anatomical challenges in urgent complex endovascular aortic repair. Ann Vasc Surg. (2022) 79:441.e1–e7. 10.1016/j.avsg.2021.07.045 34653640

[16] Arakawa M Sumikura H Okamura H Miyagawa A Homma A Yamaguchi A. A three-dimensional biomodel of type A aortic dissection for endovascular interventions. J Artif Organs. (2022) 25(2):125–31. 10.1007/s10047-021-01294-0 34609623

[17] Qin J Wu X Li W Ye K Yin M Liu G Laser fenestration of aortic arch stent grafts for endovascular treatment of retrograde type A dissection. Int J Cardiol. (2021) 328:69–74. 10.1016/j.ijcard.2020.12.011 33340586

[18] Benrashid E Wang H Andersen ND Keenan JE McCann RL Hughes GC. Complementary roles of open and hybrid approaches to thoracoabdominal aortic aneurysm repair. J Vasc Surg. (2016) 64(5):1228–38. 10.1016/j.jvs.2016.04.022 27444368 PMC5441846

[19] Huang B Yuan D Zhao J Ma Y. Hybrid treatment of a thoracoabdominal aortic aneurysm in China: report of the first successful case. Surg Today. (2012) 42(12):1219–24. 10.1007/s00595-012-0164-2 22476695

[20] Simmons MN Schreiber MJ Gill IS. Surgical renal ischemia: a contemporary overview. J Urol. (2008) 180(1):19–30. 10.1016/j.juro.2008.03.022 18485395

[21] Papadimas E Tan YK Qi Q Ng JJ Kofidis T Teoh K Left heart bypass versus circulatory arrest for open repair of thoracoabdominal aortic pathologies. ANZ J Surg. (2020) 90(12):2434–40. 10.1111/ans.16287 32935430

[22] Bickerstaff LK Pairolero PC Hollier LH Melton LJ Van Peenen HJ Cherry KJ Thoracic aortic aneurysms: a population-based study. Surgery. (1982) 92(6):1103–8. 10.1055/s-0029-1245288 7147188

[23] LeMaire SA Price MD Green SY Zarda S Coselli JS. Results of open thoracoabdominal aotic aneurysm repair. Ann Cardiothorac Surg. (2012) 1:286–92. 10.3978/j.issn.2225-319X.2012.08.16 23977510 PMC3741780

[24] Trimarchi S Tsai T Eagle KA Isselbacher EM Froehlich J Cooper JV Acute abdominal aortic dissection: insight from the international registry of acute aortic dissection (IRAD). J Vasc Surg. (2007) 46(5):913–9. 10.1016/j.jvs.2007.07.030 17980278

[25] Erbel R Aboyans V Boileau C Bossone E Bartolomeo RD Eggebrecht H 2014 ESC guidelines on the diagnosis and treatment of aortic diseases: document covering acute and chronic aortic diseases of the thoracic and abdominal aorta of the adult. The task force for the diagnosis and treatment of aortic diseases of the European society of cardiology (ESC). Eur Heart J. (2014) 35(41):2873–926. 10.1093/eurheartj/ehu281 25173340

[26] Zlatanovic P Dragas M Cvetkovic S Dimic A Mitrovic A Vujcic A Open surgical treatment of acute spontaneous isolated abdominal aortic dissection. Ann Vasc Surg. (2021) 74:525.e13–e21. 10.1016/j.avsg.2021.02.035 33836227

[27] Coselli JS Bozinovski J LeMaire SA. Open surgical repair of 2286 thoracoabdominal aortic aneurysms. Ann Thorac Surg. (2007) 83:S862–4; discussion: S890–2. 10.1016/j.athoracsur.2006.10.088 17257942

[28] Conrad MF Crawford RS Davison JK Cambria RP. Thoracoabdominal aneurysm repair: a 20-year perspective. Ann Thorac Surg. (2007) 83:S856–61; discussion: S890–2. 10.1016/j.athoracsur.2006.10.096 17257941

[29] Coselli JS Amarasekara HS Green SY Price MD Preventza O de la Cruz KI Open repair of thoracoabdominal aortic aneurysm in patients 50 years old and younger. Ann Thorac Surg. (2017) 103:1849–57. 10.1016/j.athoracsur.2016.09.058 27938888

[30] Tanaka A Sandhu HK Affi RO Miller CC 3rd Ray A Hassan M Outcomes of open repairs of chronic distal aortic dissection anatomically amenable to endovascular repairs. J Thorac Cardiovasc Surg. (2021) 161(1):36–43.e6. 10.1016/j.jtcvs.2019.09.083 31699416

[31] Crawford ES Snyder DM Cho GC Roehm JO. Progress in treatment of thoracoabdominal and abdominal aortic aneurysms involving celiac, superior mesenteric, and renal arteries. Ann Surg. (1978) 188:404–10. 10.1097/00000658-197809000-00016 686902 PMC1396963

[32] Coselli JS Conklin LD LeMaire SA. Thoracoabdominal aortic aneurysm repair: review and update of current strategies. Ann Thorac Surg. (2002) 74:S1881–4. 10.1016/S0003-4975(02)04139-5 12440686

[33] Coselli JS. The use ofleft heart bypass in the repair of thoracoabdominal aortic aneurysms: current techniques and results. Semin Thorac Cardiovasc Surg. (2003) 15:326–32. 10.1053/S1043-0679(03)00090-X 14710373

[34] Quinones-Baldrich WJ. Descending thoracic and thoracoabdominal aortic aneurysm repair: 15-year results using a uniform approach. AnnVasc Surg. (2004) 18:335–42. 10.1007/s10016-004-0033-6 15354636

[35] Frederick JR Woo YJ. Thoracoabdominal aortic aneurysm. Ann Cardiothorac Surg. (2012) 1:277–85. 10.1007/978-3-642-00418-6_2292 23977509 PMC3741772

[36] Cowan JA Jr Dimick JB Henke PK Huber TS Stanley JC Upchurch GR Jr. Surgical treatment of intact thoracoabdominal aortic aneurysms in the United States: hospital and surgeon volume-related outcomes. J Vasc Surg. (2003) 37(6):1169–74. 10.1016/S0741-5214(03)00085-5 12764260

[37] Crawford ES De Natale RW. Thoracoabdominal aortic aneurysm: observations regarding the natural course of the disease. J Vas Surg. (1986) 3:578–82. 10.1016/0741-5214(86)90281-8 3959256

[38] Parodi JC Palmaz JC Barone HD. Transfemoral intraluminal graft implantation for abdominal aortic aneurysms. Ann Vasc Surg. (1991) 5(6):491–9. 10.1007/BF02015271 1837729

[39] Dake MD Miller DC Semba CP Mitchell RS Walker PJ Liddell RP. Transluminal placement of endovascular stent-grafts for the treatment of descending thoracic aortic aneurysms. N Engl J Med. (1994) 331(26):1729–34. 10.1056/NEJM199412293312601 7984192

[40] Greenberg RK West K Pfaff K Foster J Skender D Haulon S Beyond the aortic bifurcation: branched endovascular grafts for thoracoabdominal and aortoiliac aneurysms. J Vasc Surg. (2006) 43:879–86. 10.1016/j.jvs.2005.11.063 16678676

[41] Han Y Zhang S Zhang J Ji C Eckstein HH. Outcomes of endovascular abdominal aortic aneurysm repair in octogenarians: meta-analysis and systematic review. Eur J Vasc Endovasc Surg. (2017) 54(4):454–63. 10.1016/j.ejvs.2017.06.027 28822680

[42] Tenorio ER Dias-Neto MF Lima GBB Estrera AL Oderich GS. Endovascular repair for thoracoabdominal aortic aneurysms: current status and future challenges. Ann Cardiothorac Surg. (2021) 10(6):744–67. 10.21037/acs-2021-taes-24 34926178 PMC8640886

[43] Maurel B Delclaux N Sobocinski J Hertault A Martin-Gonzalez T Moussa M The impact of early pelvic and lower limb reperfusion and attentive peri-operative management on the incidence of spinal cord ischemia during thoracoabdominal aortic aneurysm endovascular repair. Eur J Vasc Endovasc Surg. (2015) 49(3):248–54. 10.1016/j.ejvs.2014.11.017 25575833

[44] Kärkkäinen JM Sandri GA Tenorio ER Macedo TA Hofer J Gloviczki P Prospective assessment of health-related quality of life after endovascular repair of pararenal and thoracoabdominal aortic aneurysms using fenestrated-branched endografts. J Vasc Surg. (2019) 69(5):1356–1366.e6. 10.1016/j.jvs.2018.07.060 30714570

[45] Oderich GS Ribeiro M Hofer J Wigham J Cha S Chini J. Prospective, nonrandomized study to evaluate endovascular repair of pararenal and thoracoabdominal aortic aneurysms using fenestrated-branched endografts based on supraceliac sealing zones. J Vasc Surg. (2017) 65(5):1249–1259.e10. 10.1016/j.jvs.2016.09.038 27986479

[46] Gallitto E Faggioli G Melissano G Fargion A Isernia G Lenti M. Preoperative and postoperative predictors of clinical outcome fenestrated and branched endovascular repair for complex abdominal and thoracoabdominal aortic aneurysms in an Italian multicenter registry. J Vasc Surg. (2021) 74(6):1795–1806.e6. 10.1016/j.jvs.2021.04.072 34098004

[47] Quiñones-Baldrich WJ Panetta TF Vescera CL Kashyap VS. Repair of type IV thoracoabdominal aneurysm with a combined endovascular and surgical approach. J Vasc Surg. (1999) 30(3):555–60. 10.1016/S0741-5214(99)70084-4 10477650

[48] Bell KE Lopez AC. Hybrid repair of thoracoabdominal aneurysms: a combined endovascular and open approach. J Vasc Nurs. (2008) 26(4):101–8. 10.1016/j.jvn.2008.09.001 19022168

[49] Tanaka A Oderich GS Estrera AL. Total abdominal debranching hybrid thoracoabdominal aortic aneurysm repair versus chimneys and snorkels. JTCVS Tech. (2021) 10:28–33. 10.1016/j.xjtc.2021.08.002 34977700 PMC8691180

[50] Yang G Zhang M Muzepper M Du X Wang W Liu C Comparison of physician-modified fenestrated/branched stent-grafts and hybrid visceral debranching plus stent-graft placement for complex thoracoabdominal aortic aneurysm repair. J Endovasc Ther. (2020) 27(5):749–56. 10.1177/1526602820934466 32580618

[51] Ljungman C Wanhainen A Kragsterman B Nyman R Ericksson LG Eriksson. Propositions for refinement of the hybrid surgical technique for treatment of thoraco-abdominal aortic aneurysm. Scand J Surg. (2008) 97(2):174–7. 10.1177/145749690809700221 18575038

[52] Arnaoutakis DJ Scali ST Beck AW Kubilis P Huber TS Martin AJ. Comparative outcomes of open, hybrid, and fenestrated branched endovascular repair of extent II and III thoracoabdominal aortic aneurysms. J Vasc Surg. (2020) 71(5):1503–14. 10.1016/j.jvs.2019.08.236 31727462

[53] Riambau V Böckler D Brunkwall J Cao P Chiesa R Coppi G. Editor’s choice—management of descending thoracic aorta diseases: clinical practice guidelines of the European society for vascular surgery (ESVS). Eur J Vasc Endovasc Surg. (2017) 53(1):4–52. 10.1016/j.ejvs.2016.06.005 28081802

[54] Chiesa R Kahlberg A Mascia D Tshomba Y Civilini E Melissano G. Use of a novel hybrid vascular graft for sutureless revascularization of the renal arteries during open thoracoabdominal aortic aneurysm repair. J Vasc Surg. (2014) 60:622e30. 10.1016/j.jvs.2014.03.256 24768367

[55] Hughes GC Barfield ME Shah AA Williams JB Kuchibhatla M Hanna JM. Staged total abdominal debranching and thoracic endovascular aortic repair for thoracoabdominal aneurysm. J Vasc Surg. (2012) 56(3):621–9. 10.1016/j.jvs.2011.11.149 22575483 PMC4089876

